# Views on Structure-based design of antiviral drug candidates targeting the SARS-CoV-2 main protease by Dai et al. (2020)

**DOI:** 10.6026/97320630016435

**Published:** 2020-05-31

**Authors:** Hasanain Abdulhameed Odhar

**Affiliations:** 1 Department of pharmacy, Al-Zahrawi University College, Karbala, Iraq

## Views:

The project for the report [1] seems to be well funded and the authors had access to many modern facilities and techniques. They were able to successfully design, synthesize and evaluate two lead inhibitors of SARS-CoV-2 main protease. Interestingly, they were able to crystallize protease-inhibitor complex to visualize real chemical interaction involved. According to x-ray diffraction images, they found that the aldehyde group of synthesized inhibitors is involved in covalent bond with Mpro active site. We can appreciate this finding by noting that computational modeling approaches like docking studies have difficulties in predicting covalent bonds. The design of these two novel inhibitors may be considered a continuation for several previous attempts of structure-based design of coronavirus main protease inhibitors, for example the well-known peptide inhibitor N3. I am only concerned about the interaction between lead inhibitors and water molecules in crystallization images. These water molecules may be active site reserved water or merely the result of crystallization process. It would be interesting to prepare several derivatives of these two lead inhibitors and then establish a structure-activity relationship study. This seems to be crucial for elucidating the structure of core scaffold.
